# Supplementary material for: What Is the Relationship between Trunk Control Function and Arm Coordination in Adults with Severe-to-Moderate Quadriplegic Cerebral Palsy?
Source: Int J Environ Res Public Health. 2022 Dec 22;20(1):141. doi: 10.3390/ijerph20010141 (PMC9819854; doi:10.3390/ijerph20010141)
Supplement: Supplementary file 1 [file ijerph-20-00141-s001.zip › ijerph-2076691-supplementary.pdf]

Supplementary Materials

What is the relationship between trunk control function and arm coordination in adults with severe-to-moderate quadriplegic cerebral palsy?

María Isabel Cornejo, Alba Roldan, Raul Reina

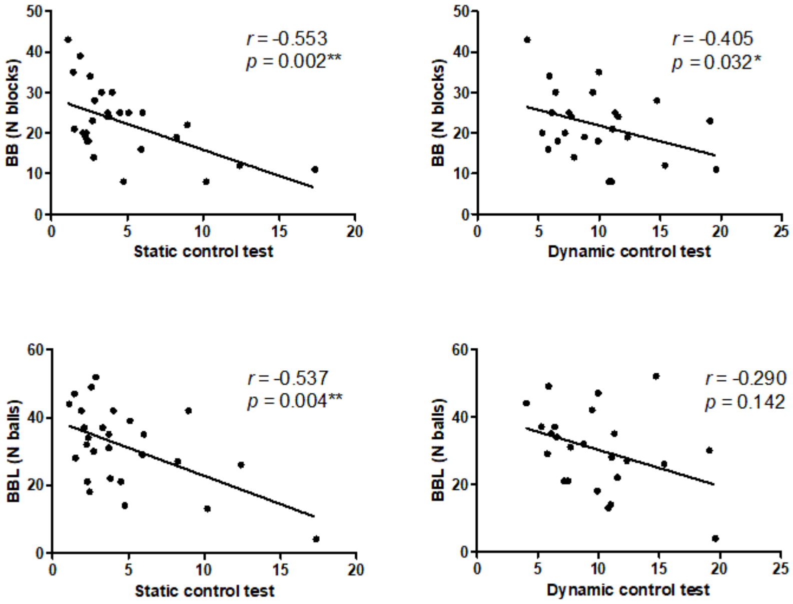

Figure S1. Pearson product correlation between manual dexterity and the dynamic and static control tests. BB = Box and Block test; BBL = Box and Ball test.

What is the relationship between trunk control function and arm coordination in adults with severe-to-moderate quadriplegic cerebral palsy?

María Isabel Cornejo, Alba Roldan, Raul Reina

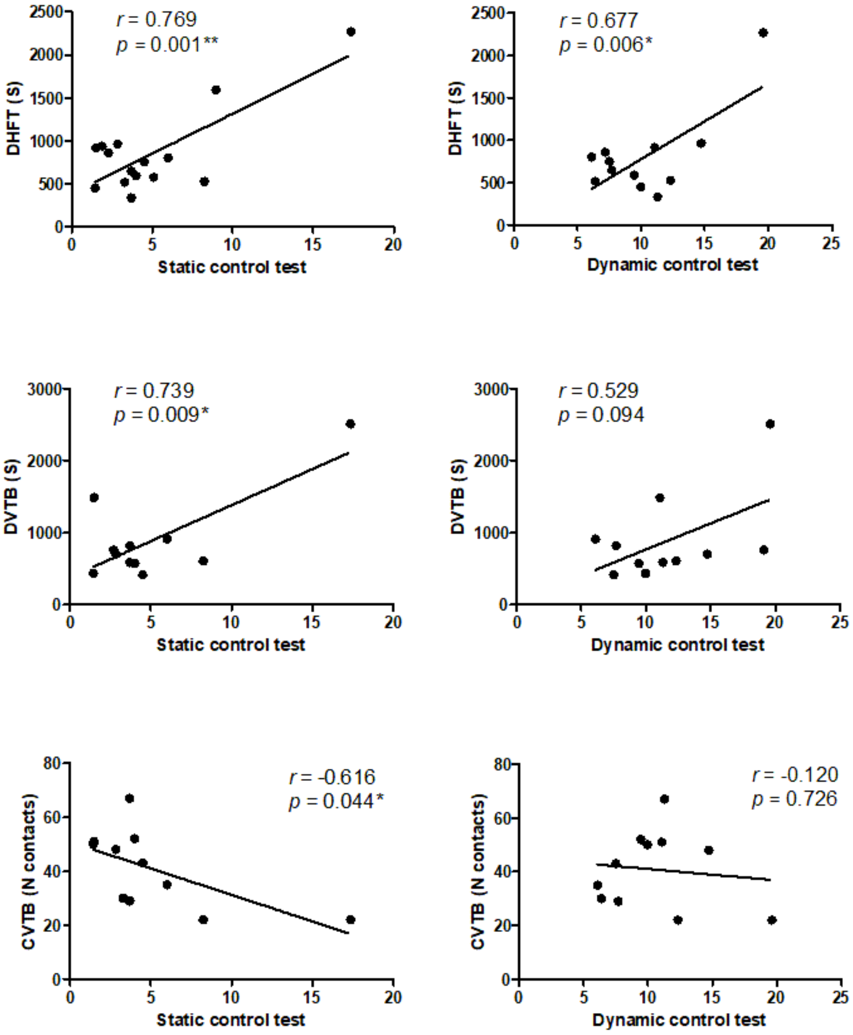

Figure S2. Pearson product correlation between intra-limb coordination and static and dynamic trunk control tests. DHFT = Discrete Horizontal Finger Tapping test; DVTB = Discrete Vertical Tapping with Ball test; CVTB = Continuous Vertical Tapping with Ball test.
